# Diffuse low-grade glioma: What is the optimal linear measure to assess tumor growth?

**DOI:** 10.1093/noajnl/vdae044

**Published:** 2024-03-27

**Authors:** Thomas Dos Santos, Jeremy Deverdun, Thierry Chaptal, Amélie Darlix, Hugues Duffau, Liesjet Elisabeth Henriette Van Dokkum, Arthur Coget, Mathilde Carrière, Eve Denis, Margaux Verdier, Nicolas Menjot de Champfleur, Emmanuelle Le Bars

**Affiliations:** Department of Neuroradiology, Montpellier University Medical Center, Montpellier, France; Department of Neuroradiology, Montpellier University Medical Center, Montpellier, France; I2FH, Institut d’Imagerie Fonctionnelle Humaine, Department of Neuroradiology, Montpellier University Medical Center, Montpellier, France; Department of Neuroradiology, Montpellier University Medical Center, Montpellier, France; I2FH, Institut d’Imagerie Fonctionnelle Humaine, Department of Neuroradiology, Montpellier University Medical Center, Montpellier, France; Department of Medical Oncology, Institut Régional du Cancer de Montpellier (ICM), University of Montpellier, Montpellier, France; Institute of Functional Genomics, University of Montpellier, CNRS, INSERM, Montpellier, France; Department of Neurosurgery, Gui de Chauliac Hospital, Montpellier University Medical Center, Montpellier, France; Institute of Functional Genomics, INSERM 1191, University of Montpellier, Montpellier, France; Department of Neuroradiology, Montpellier University Medical Center, Montpellier, France; I2FH, Institut d’Imagerie Fonctionnelle Humaine, Department of Neuroradiology, Montpellier University Medical Center, Montpellier, France; Department of Neuroradiology, Montpellier University Medical Center, Montpellier, France; Department of Neuroradiology, Montpellier University Medical Center, Montpellier, France; Department of Neuroradiology, Montpellier University Medical Center, Montpellier, France; Institute de Recherche en Cancerologie Montpellier, Montpellier University, INSERM, Montpellier, France; Department of Neuroradiology, Montpellier University Medical Center, Montpellier, France; I2FH, Institut d’Imagerie Fonctionnelle Humaine, Department of Neuroradiology, Montpellier University Medical Center, Montpellier, France; Laboratoire Charles Coulomb, University of Montpellier, Montpellier, France; Department of Neuroradiology, Montpellier University Medical Center, Montpellier, France; I2FH, Institut d’Imagerie Fonctionnelle Humaine, Department of Neuroradiology, Montpellier University Medical Center, Montpellier, France

**Keywords:** low-grade glioma, longitudinal growth quantification, linear assessment, MRI, neuro-oncology

## Abstract

**Background:**

Radiological follow-up of diffuse low-grade gliomas (LGGs) growth is challenging. Approximative visual assessment still predominates over objective quantification due to the complexity of the pathology. The infiltrating character, diffuse borders and presence of surgical cavities demand LGG-based linear measurement rules to efficiently and precisely assess LGG evolution over time.

**Methods:**

We compared optimized 1D, 2D, and 3D linear measurements with manual volume segmentation as a reference to assess LGG tumor growth in 36 patients with LGG (340 magnetic resonance imaging scans), using the clinically important mean tumor diameter (MTD) and the velocity diameter expansion (VDE). LGG-specific progression thresholds were established using the high-grade gliomas-based RECIST, Macdonald, and RANO criteria, comparing the sensitivity to identify progression/non-progression for each linear method compared to the ground truth established by the manual segmentation.

**Results:**

3D linear volume approximation correlated strongly with manually segmented volume. It also showed the highest sensitivity for progression detection. The MTD showed a comparable result, whereas the VDE highlighted that caution is warranted in the case of small tumors with multiple residues. Novel LGG-specific progression thresholds, or the critical change in estimated tumor volume, were increased for the 3D (from 40% to 52%) and 2D methods (from 25% to 33%) and decreased for the 1D method (from 20% to 16%). Using the 3D method allowed a ~5-minute time gain.

**Conclusions:**

While manual volumetric assessment remains the gold standard for calculating growth rate, the 3D linear method is the best time-efficient standardized alternative for radiological evaluation of LGGs in routine use.

## Lay Summary

Low-grade gliomas are brain tumors that arise from the brain itself. Magnetic resonance imaging (MRI) is often used to monitor tumors because they can grow in unpredictable ways. Measuring tumor volume is the best method to accurately determine size, but it is difficult and time-consuming to do this, particularly after surgery. The authors in this study aimed to find a quicker but still reliable way to estimate tumor volume after surgery. To do this, they compared volume calculations to simpler linear measurements of tumors in one, two-, and three-dimensions using MRI scans from 36 patients. Their results showed that measuring the tumor in three dimensions most closely matched the tumor volume calculations, although it was not perfect.

**Key Points**

1. 3D linear assessment is the best time-efficient quantitative alternative to manual segmentation in diffuse low-grade glioma volume estimation.

2. 3D linear assessment is the most sensitive to small changes in diffuse low-grade glioma volume over time.

**Importance of the Study**

The follow-up of LGG volume change is primordial, as the detection of small variations might help in therapeutic management. The most precise way is by means of manual segmentation, yet this is time-consuming. Therefore, most neuroradiologists simply use approximate visual inspection. Here we showed that the best alternative, allowing for a precise quantification while being time efficient, is the 3D linear assessment method. It showed both the best correlation with manual segmentation, as well as the highest sensitivity to detect small changes in tumor size.

WHO grade 2 low-grade gliomas (LGGs)^1^ are primary malignant heterogeneous brain tumors and represent 20% of all gliomas.^2^ They are quantified as slow-growing and infiltrative lesions, that primarily occur in young adults.^3–5^ Despite great individual variability, the natural history of LGGs is marked by an unavoidable malignant transformation into a higher grade. Awake surgery with electrostimulation allowing maximal tumor resection while preserving eloquent areas, is currently the first-line treatment, which has increased overall survival while preserving patients’ quality of life.^6^ The decision to take therapeutic action, whether surgery or adjunctive chemo or radiotherapy, is dependent on various factors, including the estimated tumor growth and the grade of malignancy. Both can be evaluated with magnetic resonance imaging (MRI) FLAIR-weighted imaging, that is, currently the gold standard for diagnosis and clinical follow-up.^7,8^

The most used method to estimate tumor growth is qualitative visual inspection despite European recommendations to use the RANO-LGG criteria.^9^ A recent study highlighted that only 19.4% of MRI interpretations included quantitative tumor measurements by radiologists.^10^ Although at the end of the 20th century, neither visual, area, or volume changes confidently predicted clinical outcomes,^11^ we now know that subjective visual assessment fails to recognize small variations over time, leading to delayed progression detection.^10,12^ Therefore, various experts advocate for the routine use of objective measurements and have been doing so for the last 10 years.^7,12^ Given the irregular and infiltrative nature of LGGs, the most precise estimation of tumor volume is obtained by manual segmentation, but this is a tedious and time-consuming exercise, and therefore difficult to apply in daily practice.^13^ Actually, artificial intelligence tools for automated LLG volume segmentation are under development.^14,15^ Thus, pending their availability, linear measurements are considered as the best compromise for the radiological volume evaluation of LGGs in clinical practice. More specifically, Gui and colleagues recommended the use of 3D linear measurements for LGG tumor size evaluation, based on a study with LLG patients evaluated before surgical intervention.^10^

However, following the current guidelines most patients undergo surgical intervention as the first line of treatment. The presence of a cavity and/or multiple diffuse residues increases the complexity of the radiological volume evaluation.^16^ Therefore, we aim to determine which linear measurement method provides the optimal compromise for time-efficient radiological assessment of LGGs after surgical intervention in daily practice, by comparing one-dimensional (1D), two-dimensional (2D), and three-dimensional (3D) linear measurements with manual volume segmentation in the follow-up of LGGs. We expect that, given the anisotropic growth of LGGs along white matter fibers, the 3D method will be most likely to capture small variations in tumor growth. When linear measurements are performed in an optimal manner, the 3D linear method might also allow for reliable calculation of the mean tumor diameter (MTD) and the related velocity diameter expansion (VDE) rate, important for clinical follow-up.^3^

## Materials and Methods

### Participants

In this observational multicentric study 36 patients (age 40 + -7,2; 19 male) who underwent surgery with a histological diagnosis of LGG were retrospectively included from the SPECIFY database (longitudinal patient follow-up between 2009-2020, approved by the local ethical committee: NCT04346472_UF9647, Montpellier University Hospital), resulting in a total of 340 MRI scans and a minimal follow-up time of 14 months, with imaging every 3 to 6 months. Inclusion criteria were: Age > 18, histological evidence of LGG according to the revised 2016 WHO classification, and MRI data without artifacts. Images < 72 hours post-surgery were excluded from analysis due to interference with postsurgical changes (enhancement, edema, and ischemia). Procedures were compliant with the Declaration of Helsinki. All participants gave informed consent by non-opposition.

### Imaging

MRI scans were acquired on either 1.5-Tesla or 3-Tesla scanner with a phase array multi-channel head coil (16 or 32 channels) or head neck coil (20 or 64 channels). Pre- and post-gadolinium T1 weighted images were analyzed to evaluate eventual malignant transformation. Possible variations due to head positioning in the MRI were limited by using automatic plane acquisitions (axial, coronal, and sagittal) according to the reference plane through the anterior–posterior commissures.^17^ Small variations in head positions could not be fully excluded, especially in the case of large tumors/cavity that challenged the automatic identification of the chosen landmark.^13,14^ Nevertheless, subsequent quality control assessment was performed to assure correct positioning.

### Protocol

**Figure 1.**
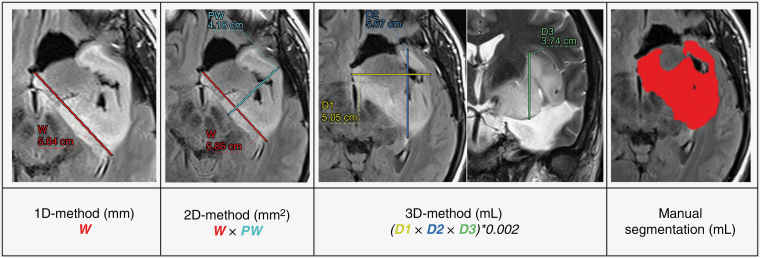
Linear measurement and manual segmentation examples.

The linear measurements included 5 diameters as illustrated in Figure 1. The tumor width (W) was defined as the longest diameter in any direction, whereas the perpendicular width (PW) was the largest diameter perpendicular to W in the same plane. The first diameter (D1) was defined as the maximal transversal distance, the second diameter (D2) as the maximal antero–posterior distance, and the third diameter (D3) as the maximal height. W, PW, D1, and D2 were extracted from the 2D or 3D FLAIR in the axial-oblique plane of acquisition and D3 was extracted from the T2-weighted or 3D FLAIR images in the coronal plane.

The linear measurements were performed by one investigator (TDS) with digital calipers on the PACS workstation. Two independent investigators (MV, TC) performed manual segmentation on FLAIR images using MRIcron (v1.0.20190902). To determine intra- and interrater variability, linear measurements were repeated at least 6 months later by 2 senior neuroradiologists (MC, ED) on a subset of 36 randomly selected MRIs (10% of the total population). Measurement completion time was systematically recorded.

The linear measurements were used to estimate tumor size. One-dimensional (1D) tumor size estimation was based on W, two-dimensional (2D) tumor size estimation on the product of W and PW, and three-dimensional (3D) tumor size estimation on an ellipsoidal volume approximation following. 3D=(D1×D2×D3)/2^3,7^: The presence of multiple separate lesion residues around the postsurgical cavity challenges linear measurements. Because of the lack of founded recommendations in LGGs, we established the following rules to increase reproducibility: (1) W and PW had to remain within tumor tissue as much as possible, or cavity crossing had to be maximally avoided and (2) in case of multiple residues, measurements were performed on the largest portion, excluding small and ill-defined lesions in accordance with the RECIST and the RANO criteria.^18,19^

Manual segmentation was performed by outlining the lesion on each axial plane of the native FLAIR image. Volume estimation (V) was based on the concatenated volume of the outlined masks plus the corresponding gap in between each axial plane. In addition, we also calculated the MTD, important in the clinical evaluation of the velocity of diameter expansion. We calculated the MTD based on (a) the 3 diameters technique with D1, D2, and D3 and b) the manually segmented volume, following:

(a) MTD.3D=(D1×D2×D3)13
(b) MTD.V=(2×V)13

Finally, we extracted the VDE for both the MTD.3D and MTD.V, for all assessments that were at least 6 months apart without interfering therapeutic intervention.^15^ The VDE is quantified as the slope of the linear MTD growth curve over time in years. The VDE is currently the most important clinical variable with a critical threshold at 8 mm/year that signals anaplastic transformation, compared to normal LGG growth rates vary around 3 to 4 mm/year.^3^

### Response Assessment

Tumor response was assessed with the expansion thresholds defined by 3 different methods used in high-grade glioma trials^20,21^: RECIST, Macdonald, and RANO.^13,18,19^ We compared the percentage of change between a baseline scan and each follow-up scan. The baseline scan was defined as the first MRI between 72 hours and 2 years after surgery to evaluate tumor progression, or in case of the evaluation of response to chemotherapy as the MRI prior to chemotherapy onset. Scan exclusion criteria were: a non-measurable tumor (<10 mm for 1D, < 100 mm² for 2D, and < 0,5 mL for 3D tumor size estimation), a first postoperative MRI baseline scan over 2 years post-surgery, and a post-surgery scan without follow-up. We identified 55 baseline and 230 follow-up scans. Fifty-five scans were excluded (including 36 presurgery scans). Tumor response was classified into 2 categories: (1) progression, represented by ≥ 20% linear increase for the 1D, a ≥ 25% increase for the 2D, and a ≥ 40% increase for the 3D tumor size estimation as well as for the manually segmented volume, following the RANO criteria and (2) non-progression, including partial response and stabilization below the predefined thresholds.

### Statistical Analysis

Intra- and interrater variability was quantified with Lin’s concordance correlation. The agreement between linear and segmented tumor size estimations were evaluated with a Pearson’s correlation. The Lin’s concordance correlation and the Bland-Altman correspondence were determined between the 3D tumor size estimation and the manually segmented volume, as well as between the MTD.3D – MTD.V and VDE.3D – VDE.V.

A cross-tabulation analysis between responders (ie, with significant linear tumor size increase following the RANO criteria based on the percentage of change) and non-responders was performed for each linear measurement. The classified response of the manually segmented volume was used as “golden standard.” We obtained the sensitivity, specificity, positive predictive value, negative predictive value (NVP), and the likelihood ratios for each linear measurement method. Sensitivity/specificity ROC curves were used to evaluate their classification performance, and to determine the optimal area under curve (AUC) with the corresponding optimal threshold to identify responders based on our data.

Statistical threshold was set at *P* < .05, two-sided. Analyses were performed with MedCalc statistical software (v18).

## Results

### Population Characteristics

A total of 340 MRI scans were analyzed in 36 patients (age 40 + −7,2 at diagnosis; 19 male) with a histological diagnosis of LGG. All patients were initially treated with surgery, consequently, all patients presented a surgical cavity. During our follow-up 61% had one or more repeated surgeries, 56% received adjuvant chemotherapy and 22% received radiotherapy. Patient characteristics are summarized in Table 1.

**Table 1.** Population Characteristics

Characteristics	Patients (*n* = 36)
Age—median, years (range)	40 (28–62)
Male sex—no. (%)	19 (53%)
Initial symptoms—no. (%)	
Seizure	26 (72%)
Neurological deficit	6 (17%)
Cognitive impairment	7 (19%)
Headache	4 (11%)
Asymptomatic	6 (17%)
Histology—no. (%)	
Astrocytoma	3 (8%)
Oligodendroglioma	15 (42%)
Oligo-astrocytoma	18 (50%)
Hemisphere involved–%	
Right	44%
Left	56%
Tumor location–%	
Frontal	75%
Insula	44%
Temporal	42%
Parietal	11%
Cingulate	6%
Occipital	0%
MRIs/ patient—median (range)	9 (3 – 17)
MRI follow-up time/ patient—mean, months (range)	53 (14 – 100)
Malignant transformation—no. (%)	11 (31%)
Treatments—no. (%)	
Surgery	36 (100%)
Repeated surgeries	22 (61%)
Chemotherapy	20 (56%)
Radiotherapy	8 (22%)

Histological diagnosis according to the WHO 2007 and 2016 classifications.

Malignant transformation was defined by the appearance of T1-weighted signal enhancement and confirmed by subsequent follow-up imaging or histological analysis in case of repeated surgery.

### Measurement Reproducibility

The intra-rater concordance of the linear measures was generally stronger than the interrater concordance (W: 0.948 vs. 0.765, PW: 0.942 vs. 0.831, D1: 0.912 vs. 0.742, D2: 0.902 vs. 0.698), except for the D3 height that showed comparable intra/interrater concordance: 0.873 versus 0.911. The intra-rater concordance for 1D, 2D, and 3D tumor size estimations was consequently also stronger than the inter-rater concordance (1D: 0.948 vs. 0.765, 2D: 0.953 vs. 0.831, 3D: 0.924 vs. 0.774). The manual volume segmentation showed the highest interrater correspondence (0.973), confirming its status as the “gold standard.” The median assessment times were 20s (1D), 28s (2D), 50s (3D), and 380s (V).

### Correlation Between Measurement Methods

**Figure 2.**
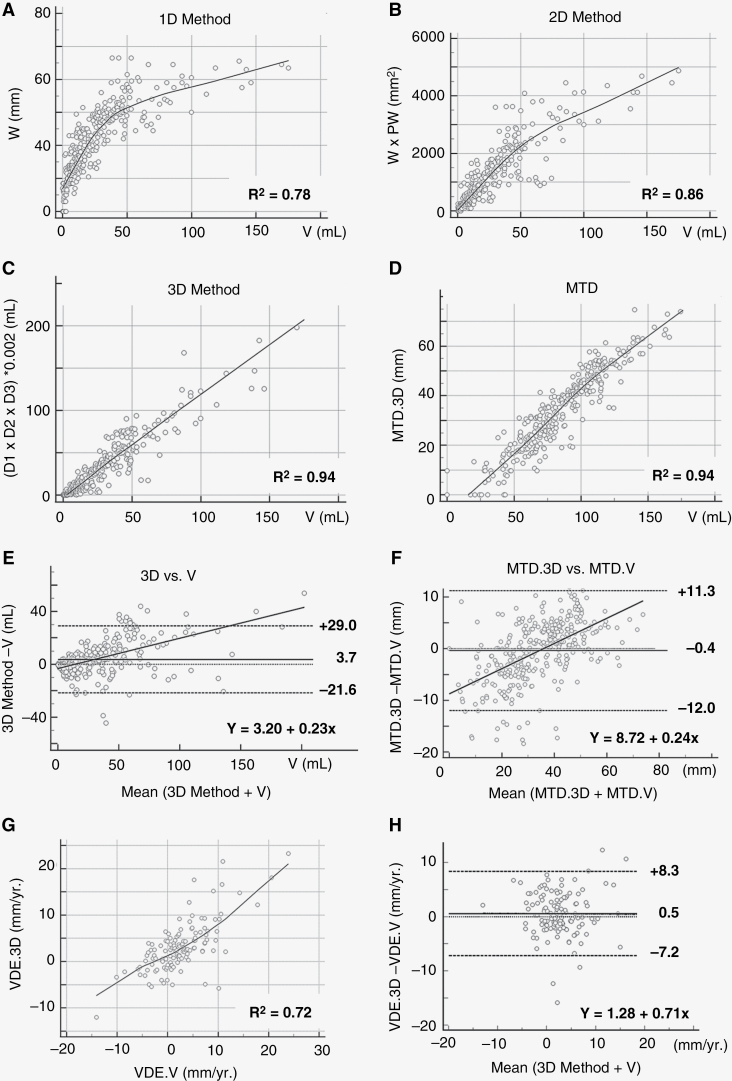
Tumor size estimation comparisons between linear measurements and manual segmented tumor volume. (A–C) Correlation between the segmented volume (V) and each linear method (A: 1D-, B: 2D-, and C: 3D-method). (D) Correlation between tumor size estimation based on the mean tumor diameter (MTD) extracted from the 3D method (MTD.3D), and the segmented volume (MTDV.V). (E–F) Bland-Altman plot evaluation of the concordance between E: 3D tumor size estimation and the segmented volume (V), and (F) MTD.3D and MTD.V. G: correlation velocity diameter expansion (VDE).3D and VDE.V. (H) Bland-Altman concordance plot between the VDE.3D and the VDE.V. R²: Pearson’s correlation coefficient. mm, millimeters; mL, milliliters.

The 3D linear tumor size estimation showed the strongest correlation with the manually segmented volume (3D: *r* = 0.94, 95% IC: [0.93–0.95]), whereas the 1D and 2D tumor size estimations only showed weak correlation with V (1D: 0.78, 95% IC: [0.74–0.82], 2D: 0.86, 95% IC: [0.83–0.88]). The MTD.3D correlated strongly with MTD.V (*r* = 0.94, 95% IC: [0.92–0.95]) with a moderate Lin’s concordance coefficient (LCC: 0.91, 95% IC: [0.89–0.92]; Figure 2A-D). Imposing a minimal MTD difference to exclude measurement errors did not further improve the concordance (Supplementary Figure 3).

The Bland-Altman analysis showed a systematic small overestimation of tumor size with the 3D estimation compared to V, yet with large upper and lower boundaries of agreement (mean: 3.7 mL, IC95% [−21.6–+29 mL]). A similar tendency was observed when comparing MTD.3D with MTD.V, with a mean overestimation of 0.4 mm (IC95% [−12–+113 mm]; Figure 2E-F). The optimal concordance between MTD.3D and MTD.V was observed for tumors with an MTD around 35 mm.

The mean VDE.3D was 3.1 mm/year (IC95% [−12 t+23 mm/year]) and the mean VDE.V was 2.6 mm/year (IC95% [−14–+24 mm/year]). The correlation between both VDE was limited (*r* = 0.72 IC 95% [0.62–0.78]). The Bland-Altman analysis showed a constant overestimation of tumor expansion by the VDE.3D of 0.5 mm/year, with a large 95% confidence interval [−7.2 to 8.3 mm/year], corresponding to the low correlation.

### Impact of MTD Precision on VDE Estimation.

On the VDE correlation plot (Figure 2G) a turning point was identified. That is, for VDE.V values below 5 mm/year the estimated VDE.3D was lower in comparison, whereas for values over 5 mm/year the estimated VDE.3D was higher. A detailed analysis confirmed that in 23.1% of the cases (all tumors with a MTD below 35 mm), the VDE.3D indicated a tumor regression, whereas the VDE.V signaled progression. To evaluate whether these discordant findings result from a measurement error in MTD, we first divided our population into two subgroups, based on the MTD bland-Altman analysis showing that the optimal concordance was between MTD.V and MTD.3D was found for a mean MTD of 35 mm (Figure 2F). This confirmed that small tumors (MTD.V < 35 mm), had a low VDE Lin’s concordance (LCC = 0.55, *n* = 67), with a large mean difference (0.9 mm/year) that was impacted by the size of the VDE. In contrast, large tumors with a MTD.V > 35 mm (LCC = 0.78, *n* = 67) had a mean VDE.V versus VDE.3D difference of 0.2 mm/year, which was only minimally impacted by the rate of the VDE (see supplementary Figure 4). Second, we performed a simulation to evaluate whether imposing minimal and maximal MTD boundaries improved the VDE concordance. This was not the case. Third, we evaluated the impact of a minimal absolute difference in MTD (ΔMTD) on VDE concordance using Bland-Altman plots, again no impact was found. And finally, a simulation evaluating the impact of minimal and maximal ΔMTD on the VDE concordance demonstrated that varying the lower limit had a stronger impact on the VDE concordance, with a first peak concordance identified at a ΔMTD of 4mm (LCC = 0.74, true for 20% of the data) and an optimal concordance peak (LCC = 0.99, true for 4% of the data) at a ΔMTD of 10mm (see Supplementary Figures 5 and 6).

### Assessment of Progression Among the Different Methods

Radiological progression of the manually segmented tumor volume was observed in 102 out of the 230 follow-up MRI scans in 27 patients. The results of the cross-tabulation analysis evaluating the performance of the linear tumor size estimation methods to identify radiological progression as defined by the RANO criteria are presented in Table 2. Of the linear methods, the 1D method showed the highest specificity, yet the lowest sensitivity. In contrast, the 3D method showed the highest sensitivity, with the lowest specificity of the three methods. Of the 3D-diameter-based measures, the MTD.3D was comparable to the 3D linear assessment as expected, yet with a slightly higher sensitivity and slightly lower specificity and AUC value.

**Table 2.** Classification Capacity

Parameters	1D	2D	3D	MTD.3D
Nr.	230	230	230	230
AUC	0.830	0.843	0.834	0.827
Sensitivity	71.6%	80.4%	86.3%	87.3%
Specificity	94.5%	88.3%	80.5%	78.1%
PPV	91.3%	84.5%	77.9%	76.1%
NPV	80.7%	85.0%	88.0%	88.5%
LR+	13.1	6.9	4.4	4.0
LR-	0.3	0.2	0.2	0.2

Quantifying the capacity of each linear method (1-dimension 1D, 2-dimensions 2D and 3-dimension 3D) and diameter measure (MTD.3D), based on the 3D volume approximation to classify patients as responders or non-responders to treatment-induced changes in the estimated tumor size over time, following the RANO criteria of progression for each measurement.

AUC, area under the curve; PPV, positive predictive value; NPV, negative predictive value; LR+, positive likelihood ratio; LR-, negative likelihood ratio.

**Figure 3.**
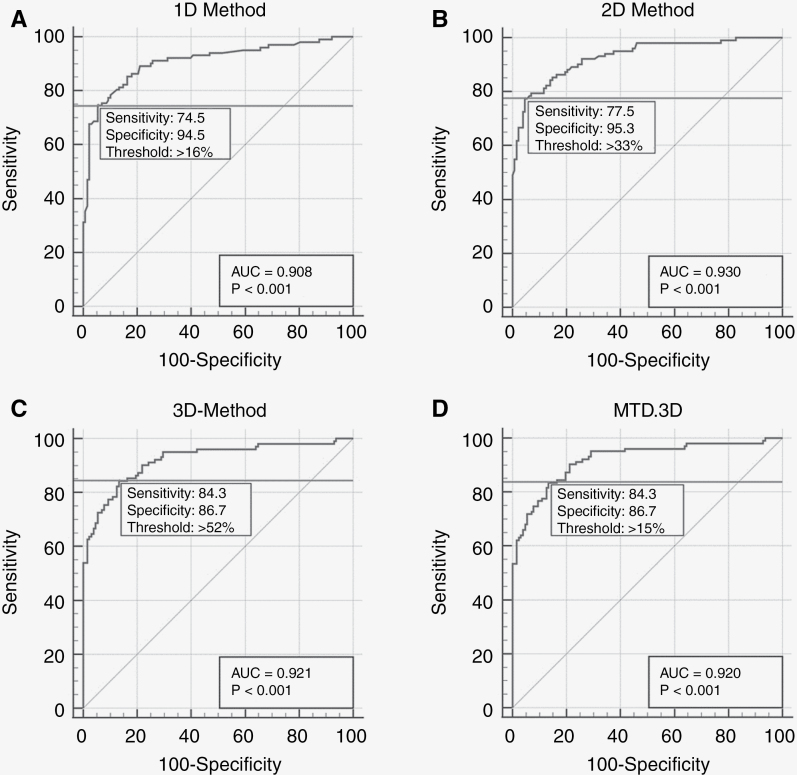
LGG progression threshold identification: the percentage of estimated tumor size change required to identify tumor progression, specific to the LGG population. Each ROC curve shows the optimal area under the curve threshold (horizontal line) with the related sensitivity and specificity values. The change in segmented manual volume was used as ground truth. (A) 1D-method, new threshold > 16%, sensitivity 74.5%, specificity 94.5%. (B) 2D-method, new threshold > 33%, sensitivity 77.7%, specificity 95.3%. (C) 3D-method, new threshold > 52%, sensitivity 84.3%, specificity 86.7%. (D) Mean tumor diameter (MTD) from 3D-method (MTD.3D), new threshold > 15%, sensitivity 84.3%, specificity 86.7%. Statistical significance was set at: *P* < .001.

Using the AUC to calculate the optimal sensitivity/specificity thresholds for each linear method based on LGG data, allowed to improve the sensitivity of the 1D method by lowering the progression threshold from 20% change (RANO criterion) to 16% change and the specificity of the 2D and 3D methods by increasing the progression thresholds from 25% (RANO criterion) to 33% (2D) and 40% (RANO criterion) to 52% (3D; Figure 3).

## Discussion

The unavailability of automated volume segmentation systems in clinical practice imposes the use of linear measurements for radiological assessment of LGGs, as manual volume segmentation is time-consuming. However, there is no consensus on the optimal method,^12^ especially in the presence of a postsurgical cavity. We compared each linear measurement method and its correlation with the manually segmented “true tumor volume” and their capacity to estimate tumor progression.

First of all, linear evaluation is indeed much quicker with assessment times below one minute, compared to manual segmentation which takes about 6 minutes. As expected, we observed a moderate correlation between linear measurements and the manually segmented “true tumor volume,” highlighting the limited capacity of linear measurements to capture the irregular shape of LGGs. In general, the linear measurements overestimated the tumor size. That said, the ellipsoidal approximation of the three orthogonal diameters (3D) was closest to the true tumor size compared to the estimation based on its 2D surface or its 1D length. These findings are consistent with those of Gui and colleagues (2019), who analyzed 103 MRI scans of 10 LGG patients, and who equally concluded the 3D method to have the highest correlation with true tumor size.^10^ However, they found a larger systematic overestimation, notably 15.9 mL compared to 3.7 mL. This might be explained by methodological differences, notably they (1) had a lower study strength with only 10 subjects and 103 MRI scans, (2) used T2 images rather than the better-delineated FLAIR images, and (3) had a population without surgical cavities.

Interestingly, the amount of overestimation we observed was dependent on the tumor size. Tumors with a volume over 35 mL were systematically overestimated by the 3D method, whereas especially small tumors were prone to size underestimation by the 3D method. The overestimation of tumor size for large tumors was confirmed expectation, as larger tumors tend to be more irregular, which increases the size of the ellipsoidal volume approximation.^7^ In contrast, the underestimation of tumor size for small tumors was more surprising. This may be caused by our optimized measuring guidelines. In the case of multiple residues, we measured only the largest one. This limited the overestimation of actual tumor size when residues were far apart, but induced underestimation in the case of small tumors for which residues are percentage-wise more important for the true volume. In line, small variations in diameter estimations have also a percentage-wise stronger impact on smaller than larger tumors (Supplementary Figures 1–2). Nevertheless, the optimization and standardization of the linear measurement allowed a more reliable estimation of tumor size, as evidenced by the high MTD.3D (mean = 31.7 mm) and MTD.V (mean = 31.8 mm) correlation (*r* = 0.94) that could not be further optimized, and by the fact that our values were comparable with the segmented tumor volume observed by Mandonnet et al. after surgery (31.2 mm), but not with their 3D estimation (36.4 mm).^22^

Secondly, we evaluated the capacity of linear measurements to quantify a change in tumor growth. As introduced previously, the presence of a cavity complicates this assessment. To increase reproducibility, we proposed strict rules, ie, the assessment of the largest residue in case of separate residues around the cavity and maximal avoidance of cavity crossing in any direction. Although the ROC curves showed comparable AUC values to discriminate progressive and non-progressive growth profiles for the 1D, 2D, and 3D methods, the sensitivity/specificity balance of each method was quite different. Both, the 1D and 2D methods were very specific, but not that sensitive. This means that they have a low false positive detection rate with a high negative one. Thus, when the threshold is reached, progression is almost certain (high specificity). However, one cannot conclude that no progression occurs when the threshold is not met (low sensitivity), as growth may remain unrecognized when it occurs outside of the measurement plane. So 1D and 2D measurements rather confirm that progression is happening, than predict it will occur. Of both, the 2D method is most commonly used in high-grade glioma follow-up, and has been recommended to be used in LGG follow-up as well, having as we confirmed both a higher specificity and sensitivity than the 1D method (RANO working group^9^).

However, it has previously been shown that 3D measurement of contrast-enhancing tumor volume in recurrent high-grade glioma follow-up is prognostic of survival.^16^ Here we are interested in predicting anaplastic transformation, or more specifically, to identify when is likely to occur, as to take preventive therapeutic action to postpone the anaplastic transformation. In line with the better 3D method’s prediction of survival in high-grade gliomas, we found that the 3D method showed the highest sensitivity rate to detect tumor growth in LGG. This suggests that the 3D method is the most suitable measure to capture small and early tumor growth. Early detection of tumor growth allows us to take full advantage of the benefits of iterative surgery, as surgery keeps the tumor size small and consequently limits the risk of malignant transformation, that is, higher for larger tumors.^23^ In addition, the 3D method has another advantage. It allows an estimation of the MTD, which in turn allows the quantification of the linear velocity of diameter expansion (VDE) between follow-up scans, currently estimated being the most reliable predictor of anaplastic transformation.^24^ As stated above, the MTD.3D correlated strongly with the MTD.V. However, this was not the case for the VDE. The correlation and concordance of the VDE were impacted by what we call the “residue effect”. For especially small tumors with multiple residues the MTD underestimated the true tumor size. When such tumors start growing, this generally happens more or less simultaneously in all residues. This is taken into account by manual segmentation, but not by our linear measurement method. Still, although caution is warranted in such cases, increasing VDE values within one residue can signal important tumor growth, relative to its own size, yet independently of the overall tumor volume. Interestingly, the VDE values in our population were much lower than those of Mandonnet et al.^22^ They found mean VDE values of 8.2 mm/year for the VDE.3D method and 6.2 mm/year for the VDE.V methods. These values are close to tumors approaching anaplastic transformation,^3^ whereas we observed values of 3.1 and 2.6 mm/year respectively which are closer to the values expected for LGG treated by surgery.^25^ This confirms that surgical intervention as first line of treatment, with or without adjuctive chemo or radiotherapy, allows to keep growth rates low over a prolonged period of time.

The high interrater variability, as well known in gliomas,^26^ remains the biggest challenge and strongest limitation of our work. By applying strict measurement rules, we aimed to limit variability. Using automatic algorithms to standardize measurements might allow the identification of optimal guidelines that may lower rater variability even further and improve correspondence between linear tumor size and the manually segmented volume estimations. Nevertheless, to limit the clinical impact of this variability, it would be preferable to have the same operator evaluate follow-up exams. Knowing this is unrealistic in clinical practice, we would suggest that a radiologist remeasures the baseline as well as the follow-up for higher reliability of progression estimation. This will however not lower variability related to different MRI acquisition parameters, between and within individual patient follow-ups, such as machine vendors, magnetic field strength, and FLAIR resolution protocol (Supplementary Table 1). Yet, MRI-based variability is inherent to clinical practice, where machines and sequences change and update over the longitudinal follow-up of patients with LGG. So, although the multicentric character of our study increased MRI-related variability, it also increased the generalizability of our findings.

Finally, one has to keep in mind that the current guidelines to identify tumor progression with linear measurements are solely based on high-grade glioma research.^20,21^ The proposed values of a 20% change to signal progression in 1D measurements, 25% in 2D measurements, and 40% in 3D measurements, do mathematically not correspond with the sphere model proposed by the 3D method.^20,27^ Id est., a 20% increase in one diameter of a sphere should correspond to a 44% surface and a 73% volume increase. The analysis of the sensitivity/specificity threshold with the optimal AUC, suggested that in case of LGG, the thresholds should indeed be adapted. For the 1D progression, a lower threshold should be used (16% of change rather than 20%), whereas higher thresholds should be used for 2D (33%) and 3D (>52%) change. Indeed, these values are more in correspondence with the sphere model. Note, however, that we only provide an initial step, as the ground truth for progression profile identification (a change in manually segmented volume over 40%) is still based on the RANO criteria for high-grade gliomas. Further research should be performed to optimize thresholds in the follow-up of LGGs. Having more precise data based on LGGs, with accordingly adapted thresholds, will equally allow us to go beyond the dichotomy of progression vs. non-progression and take into account “stability,” “partial response” and “minor-response” as well as prognostic parameters such as overall survival and progression-free survival rates. It might be argued that identifying progression by an absolute change in MTD (in mm) might be more appropriate to overcome the impact of tumor size. However, this also requires the identification of a minimal change threshold to be certain that the absolute change in mm is not caused by a measurement error. Based on our data a threshold of 4 mm could be expected, but this is rather speculative and merits thorough investigation. Finally, signs of malignant transformation and clinical considerations should be taken into account for a global evaluation, as the RANO working group aims to in development of new criteria for high-grade gliomas and brain metastases.^9,13^

## Conclusion

The assessment of LGGs by linear measurements is not perfect and often made difficult by the tumors’ irregular shape and infiltrative nature, especially after incomplete surgical resection. This work demonstrated that the 3D method is the best linear method for radiological assessment of LGGs, when automated volume segmentation is not available for precise tumor volume estimation. It showed the highest correlation with the manually segmented tumor volume, the highest sensitivity for early detection of progression and it allows extracting an approximation of the VDE that is currently the most precise predictor of eminent anaplastic transformation. However, one should be aware of the “residual effect” in small tumors with multiple residues, in this case, linear measurement might under-estimate the actual VDE. Future research requires standardization of measurement procedures as well as the definition of LGGs-specific progression thresholds.

## Supplementary Material

vdae044_suppl_Supplementary_Material

## Data Availability

Data are available upon reasonable request.
