# Supplementary material for: Diffuse low-grade glioma: What is the optimal linear measure to assess tumor growth?
Source: Neurooncol Adv. 2024 Mar 27;6(1):vdae044. doi: 10.1093/noajnl/vdae044 (PMC11274528; doi:10.1093/noajnl/vdae044)
Supplement: vdae044_suppl_Supplementary_Material [file vdae044_suppl_Supplementary_Material.docx]

**SUPPLEMENTARY MATERIAL**


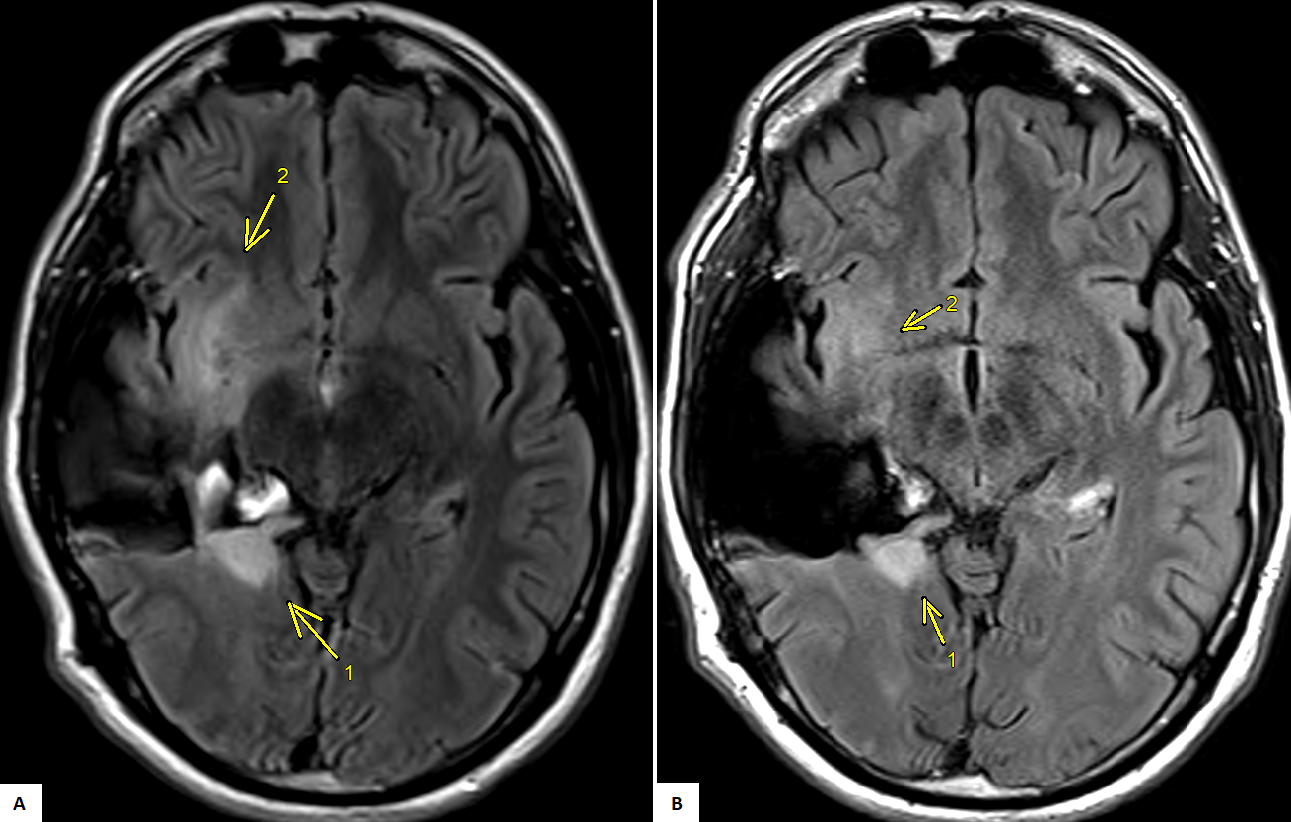


**Figure 1. Example of multiple separate residues around the surgical cavity**

37-year-old patient with a non co-deleted IDH mutated right temporal oligo-astrocytoma. A: FLAIR axial slice taken at 1 year and 9 months after surgery, showing a well-defined posterior residue indicated by arrow 1, and an ill-defined anterior residue indicated by arrow 2. B: FLAIR axial slice taken 1 year later, after 10 months of chemotherapy (Temozolomide), showing a decrease in size of the residues, predominantly in the anterior residue. Linear measurements were performed on the posterior residues.


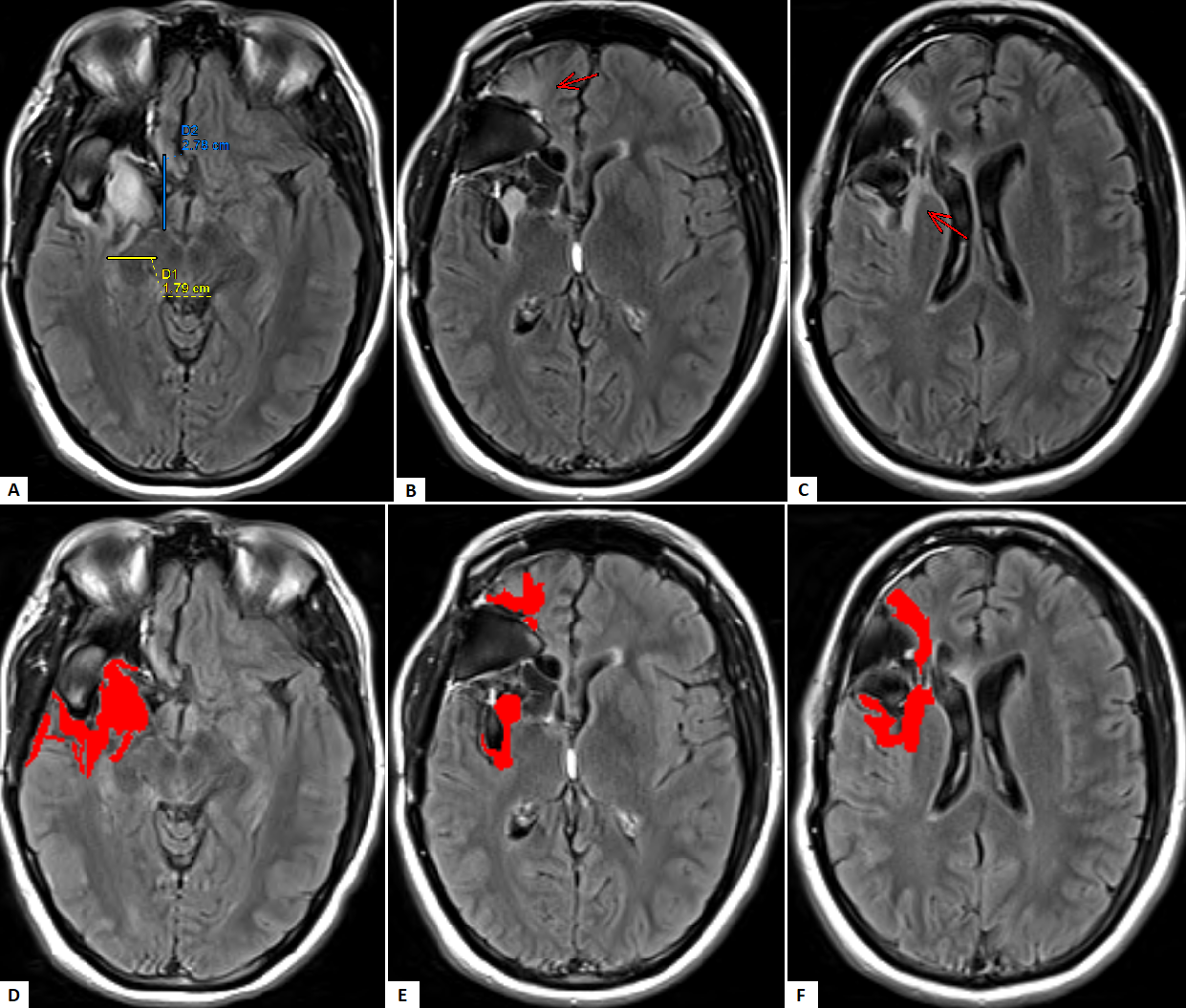


**Figure 2. Example of actual tumor volume underestimation by focussing on the largest residue only.**

MRI taken at 2-years months after surgery for a non co-deleted IDH mutated oligo-astrocytoma in a 37-year-old woman. A: FLAIR axial slice showing a residual tumour portion located at the lower and posterior part of the cavity with D1 and D2 measurements. B and C: FLAIR axial slices showing other smaller residual tumors indicated by red arrows. D, E and F: FLAIR axial sections showing the results of the manual segmentation. Underestimation of the volume by the 3D method (6 mL) compared to the volume (25 mL).


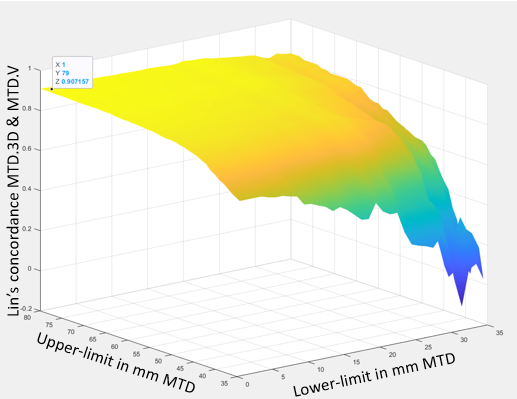


**Figure 3: Optimization of Lin’s concordance** (Z-axis) between the Mean Tumor Diameter (MTD) extracted from the linear volume approximation and the MTD based on the manual volume segmentation in mm, as a function of varying upper (Y-axis) and lower-limits (X-axis) of MTD. There is no upper limit or lower limit to be identified that allowed to improve the concordance between the two measures. This confirms the optimization of the linear measurements performed in our study.


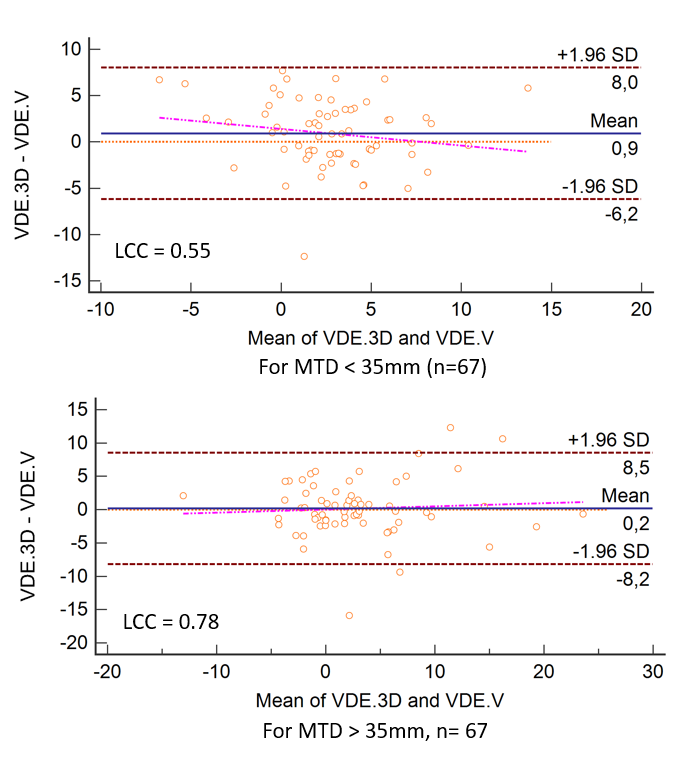


**Figure 4: Bland-Altman: Velocity Diameter Expansion concordance as a function of the mean tumor diameter (MTD).**

The figure shows the concordance between the Velocity Diameter Expansion rate based on the segmented volume or the linear volume approximation. The upper panel shows the concordance for tumors with a MTD smaller than 35mm, whereas the lower panel shows the concordance for tumors with a MTD over 35mm. A better concordance and a smaller difference in VDE is observed for larger tumors.

LCC = Lins concordance coefficient.


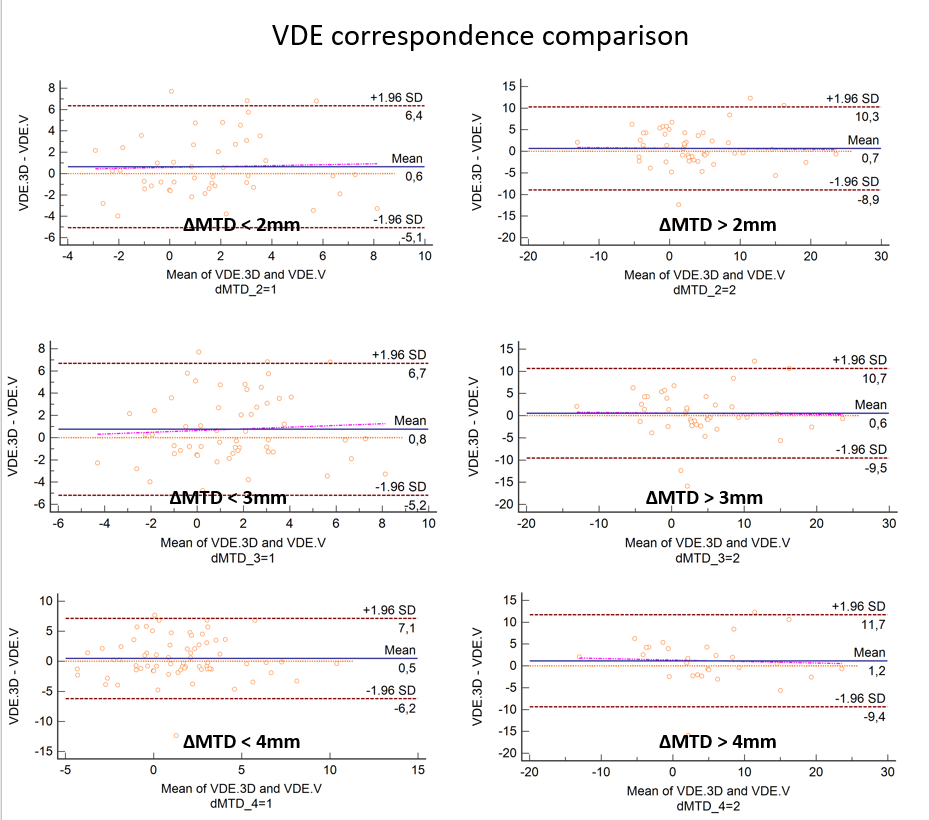


**Figure R5: Bland-Altman: Velocity Diameter Expansion (VDE) correspondence as a function of a minimal absolute difference in Mean Tumor Diameter (MTD).**

Bland-Altman plots, comparing VDE.3D estimates with the ground truth defined by manual segmentation (VDE.V) as a function of the absolute difference in diameter (deltaMTD.V in mm) of either 2mm (upper-), 3mm (middle-) and 4mm (lower-panel) between the two time-points used to calculate the VDE. No conclusions can be drawn about the minimal difference that allows to state with certainty the VDE reflects tumor growth rather instead of a measurement error.


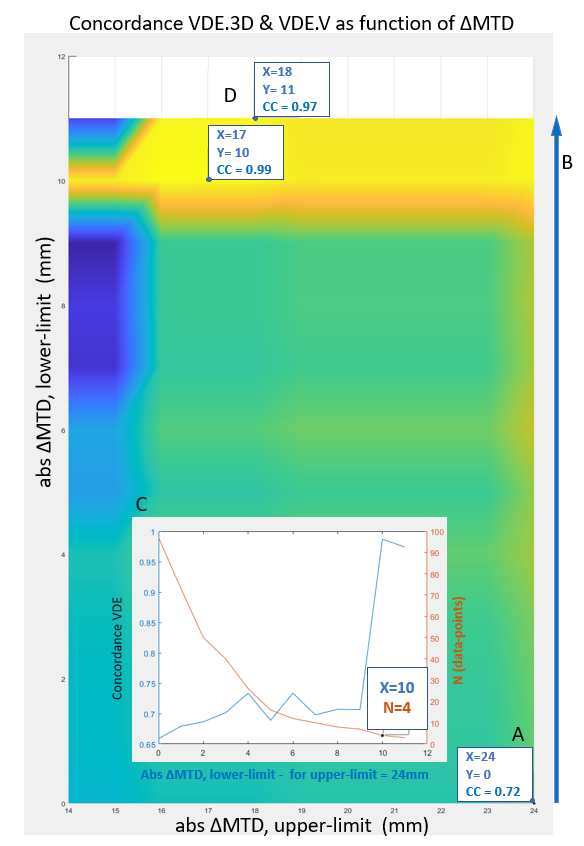


**Figure R6: Surface plot evaluating the concordance (blue = low, yellow = high) between the VDE.3D and VDE.V as a function of the absolute difference in diameter (deltaMTD.V)**, aiming to define the change in MTD.V that maximizes the concordance between VDE.3D and VDE.V. To start: A) Concordance (CC) when no upper (X=24, the maximum difference observed in our population) or lower limit (Y = 0, no change in MTD) is imposed. B). the surface plot shows that the concordance improves while increasing the lower-limit of the MTD.V (Y-axis) along the blue arrow. C) A graph of the concordance evolution (blue line) as a function of increasing lower-limit, with a peak concordance found at a lower-limit of 10mm deltaMTD.V, yet this is based on only 4 datapoints out of the 100 calculated (red line). D) identification of optimal range: a maximum concordance between VDE.3D and VDE.V (CC=0.99) is found when the deltaMTD.V is between 10 and 17mm, yet this is only true for a small proportion of the data.

**Table 1.** MRI acquisition parameters

| Acquisition parameters | Total MRI exams (n = 340) |
| --- | --- |
| Magnetic field strength – no. (%) |  |
| 1.5T | 165 (48.5%) |
| 3T | 175 (51.5%) |
| FLAIR resolution protocol – no. (%) |  |
| 2D (slice thickness: 3 – 5 mm) | 320 (94%) |
| 3D (slice thickness: 0.9 – 1.5 mm) | 20 (6%) |
| Machine vendors – no. (%) |  |
| Siemens | 322 (95%) |
| Philipps | 10 (3%) |
| GE | 8 (2%) |
